# Hispanic/Latino ethnic background and genetic ancestry in relation to atherosclerotic cardiovascular disease risk estimation: Findings from the Multi-Ethnic Study of Atherosclerosis (MESA)

**DOI:** 10.1016/j.ajpc.2025.101268

**Published:** 2025-08-22

**Authors:** Bede N. Nriagu, Karen Flores Rosario, Spencer Hansen, Priscilla Duran Luciano, Parag Joshi, Anurag Mehta, Amit V. Khera, Robert Kaplan, Tamar Sofer, Robyn L. McClelland, Carlos J. Rodriguez

**Affiliations:** a Department of Internal Medicine, New York Medical College/Metropolitan Hospital, 1901 First Avenue, New York, NY 10029, USA; b Department of Cardiology, Duke University School of Medicine, NC, USA; c Department of Biostatistics, University of Washington, Seattle, WA, USA; d Department of Cardiology, Albert Einstein College of Medicine/ Montefiore Medical Center, Bronx, NY, USA; e Division of Cardiology, University of Texas Southwestern, Dallas, TX, USA; f Department of Cardiology, VCU Health Pauley Health Center, Richmond, VA, USA; g Division of Cardiology, Brigham and Women’s Hospital, Boston, MA, USA; h Department of Medicine, Harvard Medical School, Boston, MA, USA; i Verve Therapeutics, Boston, MA, USA; j Department of Epidemiology and Population Health, Albert Einstein College of Medicine, Bronx, NY, USA; k Division of Public Health Sciences Fred Hutchinson Cancer Research Center, Seattle, WA, USA

**Keywords:** Hispanic ethnicity, Genetic ancestry, ASCVD risk estimation

## Abstract

**Background:**

Hispanics/Latinos are a heterogenous population with no validated atherosclerotic cardiovascular disease (ASCVD) risk estimation tool. We examined performance of the pooled cohort equation (PCE) across Hispanic/Latino background groups and quantiles of African, Amerindian, and European genetic ancestry.

**Methods:**

The Multi-Ethnic Study of Atherosclerosis (MESA) was used to evaluate the performance of the non-Hispanic Black (NHB) and non-Hispanic White (NHW) PCE defined by predicted to observed (P/O) ratios of 10-year ASCVD events. Risk calibration was expressed as P/O ratios and risk discrimination was assessed with Harrell’s C-statistic based on Cox models.

**Results:**

Our study included 966 Hispanics/Latinos [age 58 years at baseline (SD=9); 52 % females], comprising 504 Hispanics/Latinos of Mexican descent (MX), 284 Hispanics/Latinos of Caribbean descent (CA) and 178 Other Hispanics (O). At 10-years, there were 54 ASCVD events: MX (33); CA (13) and O (8). The PCE overestimated ASCVD risk across disaggregated Hispanic/Latino background groups. Both NHW and NHB PCE models performed best with increasing European genetic ancestry (NHW PCE: P/O ratio decreasing from 1.5 to 1.0; NHB PCE: from 2.4 to 1.5 between the 20th and 80th quantile threshold of European genetic ancestry). In contrast, PCE performance worsened with increasing African genetic ancestry (NHW PCE: P/O ratio increasing from 1.5 to 2.6; NHB PCE: from 1.5 to 2.9 between the 20th and 80th quantile threshold of African genetic ancestry).

**Conclusions:**

Disaggregating Hispanics/Latinos by background and ancestry led to variability in PCE performance with greater overestimation of ASCVD risk for those of Caribbean background and those with increasing quantiles of African genetic ancestry.

## 1 Introduction

The pooled cohort equations (PCE) constitute the basis of cardiovascular risk assessment by estimating the 10-year risk of developing a first atherosclerotic cardiovascular disease (ASCVD) event, and are widely used to guide the use of statins and hypertension treatment [1]. In the derivation of the PCE, risk estimation is only available for non-Hispanic Whites (NHW) and non-Hispanic Blacks (NHB), excluding other racial and ethnic groups such as Hispanics/Latinos, Asians and Native Americans [1].

Currently, over 63 million Hispanics/Latinos live in the United States (US) [2], making them the largest ethnic minority group in the country [3,4]. Despite Hispanics/Latinos having a higher prevalence of cardiovascular risk factors compared to NHW [3] and cardiovascular disease (CVD) being a common cause of death among them [5], no ASCVD risk estimation tool exists for this population.

Hispanic/Latino individuals are highly admixed, with varying degrees of African/European/Amerindian genetic ancestries [6] and national origins [3]. Consequently, for individuals of Hispanic/Latino background, current prevention guidelines recommend using the NHW PCE to estimate 10-year ASCVD risk, and the NHB PCE if African ancestry is known or suspected [7]. However, limited evidence exists regarding the performance of the NHW vs NHB PCE ASCVD risk estimation in Hispanic/Latino populations [8], and data evaluating the performance of the PCE across the continental genetic ancestries (African, Amerindian, and European) within this group are lacking. Moreover, the exclusion of Hispanic/Latino individuals from the original PCE derivation cohort creates a significant gap in risk prediction for this population. This underrepresentation limits the ability to evaluate the tool’s accuracy across diverse Hispanic/Latino subgroups, increasing the likelihood of risk misclassification and potentially worsening existing cardiovascular health disparities. Leveraging the Multi-Ethnic Study of Atherosclerosis (MESA), we examined whether PCE risk calibration differed among disaggregated Hispanic/Latino background groups and whether PCE performance varied based on quantiles of African, Amerindian, and European genetic ancestry.

## 2 Materials and methods

### 2.1 Study population

Briefly, MESA is a longitudinal cohort study of 6814 adults between the ages of 45 and 84, comprising 39 % non-Hispanic White, 28 % African American, 22 % Hispanic, and 12 % Chinese American who were free of any cardiovascular disease at enrollment. Participants were sampled from six US cities (Chicago, IL, Manhattan and Bronx, NY, Baltimore, MD, Los Angeles, CA, St. Paul, MN, and Forsyth County, NC) and enrolled between 2000 and 2002. The study was approved by the Institutional Review Board at each site, and all participants provided informed consent [9]. Of the 6814 participants in the MESA, Hispanics/Latinos comprised 1496 of the participants, of which 1331 were ages 45–75 at MESA exam 1 (baseline). We excluded 216 participants with diabetes mellitus. Of the remaining 1115 participants, 108 and 41 participants were excluded for using statin medications and for having low-density lipoprotein (LDL) cholesterol (LDL-C)>190mg/dL, respectively. The final analytic dataset had 966 Hispanic/Latino participants.

### 2.2 Pooled cohort equation variables

Participant’s age, sex, race and ethnicity, tobacco use, medical conditions, and current use of both prescription and nonprescription medications were collected using questionnaires [9]. Trained staff measured the participants' resting blood pressure three times using a Dinamap® automated blood pressure device, and the average of the last two readings was used for analysis. Hypertension was defined as systolic ≥140 mm Hg and/or diastolic ≥90 mm Hg (to maintain alignment with the prevailing guidelines at the time of MESA participant enrollment), or use of antihypertensive medications. Diabetes was based on self-report of physician diagnosis, fasting glucose ≥126 mg/dL, or use of insulin or oral hypoglycemic medication. Smoking status was classified as current, former, or never. Blood samples were taken by MESA-trained staff to assess lipid profiles including high density lipoprotein (HDL) cholesterol, triglycerides and total cholesterol [9]. LDL-C was calculated using the Friedewald equation [10].

### 2.3 Hispanic/Latino background groups and genetic ancestry estimation

Participants were stratified based on self‐reported heritage into three background groups: 1) Hispanics/Latinos of Caribbean descent (CA [Dominicans, Cubans, and Puerto Ricans]); 2) Hispanics/Latinos of Mexican descent (MX); and 3) Other Hispanics/Latinos (O [Central/South Americans]).

Ancestry informative markers (AIMs) were used to quantify the percentage of different genetic ancestries (African, Amerindian, and European) for a given MESA Hispanic/Latino individual. AIMs show large allele frequency differences across various populations, hence have been used in differentiating ancestry groups. Individual ancestry in MESA was estimated using 171 AIMs and individual level genotype data from 3 ancestral populations: Yoruba Nigerians, European Ancestors from Hapmap (https://www.genome.gov/10001688/international-hapmap-project), and participants from Native American population [11,12].

Individual genetic admixture proportions were determined using a Markov Chain-Monte Carlo (MCMC) approach [13,14] with the program STRUCTURE 2.1. Further details on selection of AIMs and determination of genetic ancestry among MESA Hispanics/Latinos have been described elsewhere [15,16]. Participants were further stratified into quantile thresholds of African, Amerindian, and European genetic ancestry.

### 2.4 ASCVD events adjudication

Information about ASCVD events (coronary heart disease deaths, strokes, fatal and non-fatal myocardial infarctions [MI]) was gathered by trained MESA personnel from hospital medical records, participant interviews, death certificates, autopsy reports, the National Death Index, and from interviews administered to relatives or physicians through the study follow-up calls. The MESA endpoints committee adjudicated these events through review by at least two physicians, and an analysis by computer algorithm (for MI events only) using standardized criteria to assign a final classification. Differing diagnoses between two reviewers are discussed and resolved, and a selected number of cases may be reviewed by the entire MESA Morbidity and Mortality (M&M) Committee for quality purposes. Additional details on the MESA study’s follow-up methods and ASCVD event adjudication can be found on the MESA web site at http://www.mesa-nhlbi.org.

### 2.5 Statistical analysis

We calculated the 10-year ASCVD risk of MESA Hispanics/Latinos across continental genetic ancestry (African, Amerindian, and European) and quantile thresholds of African, Amerindian, and European genetic ancestry. To account for those who were censored before 10 years follow up, one minus the 10-year Kaplan-Meier estimate of survival yielded the observed rate at 10-years. We used the mean NHW and NHB PCE risk score for each subgroup as the predicted event rate. The predicted-to-observed (P/O) ratios were subsequently calculated by dividing the predicted event rate by observed event rate. P/O ratios converging toward 1.0 were deemed as best performing, while overestimation was reflected by P/O ratio >1.0 and underestimation by P/O ratio <1.0. Analyses were performed across 1) Hispanic/Latino background groups and 2) greater than median African, Amerindian and European genetic. The median genetic ancestry thresholds were 0.049, 0.444 and 0.500 for African, Amerindian, and European, respectively. Analyses of P/O ratios were conducted on subgroups and also stratified by ASCVD risk categories: low and borderline (<7.5 %), intermediate (7.5 % to 20 %), and high (>20 %). Cox proportional hazards models were fitted on time to ASCVD with NHW or NHB PCE risk scores as the predictor. Harrell’s C-statistics were calculated as a measure of risk discrimination (to evaluate concordance between predicted and observed events from these time-to-event models and compared using a Z-test).

## 3 Results

Our study included 966 Hispanic/Latino individuals [58 years at baseline (SD=9); 52 % females], comprising 504 MX, 284 CA and 178 O. The mean (range) follow-up period was 9.1 years (0.3 - 10.1). At 10 years, there were 54 ASCVD events (Table 1). Baseline Characteristics across disaggregated Hispanic/Latinos subgroups and greater than median African, Amerindian and European genetic ancestry can be found in (Table 1). The PCE overestimated ASCVD risk across disaggregated Hispanic/Latino background groups (Fig. 1A). Across disaggregated Hispanics/Latinos background groups, the extent of overestimation varied. Using the NHB PCE, the least overestimation was observed in MX within the intermediate-risk category with a P/O ratio of 1.08. Conversely, the highest overestimation was noted among CA in the high-risk category with a P/O ratio of 2.71. In contrast, when using the NHW PCE, the least overestimation was found among O in the high-risk category, with a P/O ratio of 1.07, while the highest overestimation was noted in CA within the high-risk category, with a P/O ratio of 2.45 (Fig. 1A)**.** In each of the disaggregated Hispanics/Latinos background groups, overestimation was highest among 1) MX in the low and borderline-risk category using both the NHB PCE (P/O ratio of 2.31) and NHW PCE (P/O ratio of 1.27), 2) CA in the high-risk category using both the NHB PCE (P/O ratio of 2.71) and NHW PCE (P/O ratio of 2.45), 3) O in the low & borderline-risk category using the NHB PCE (P/O ratio of 3.09) and intermediate-risk group using the NHW PCE (P/O ratio of 2.05) (Fig. 1A)**.**

**Table 1:** Baseline Characteristics across disaggregated Hispanic/Latinos subgroups and per continental genetic ancestry (African, Amerindian and European) by adjudicated ASCVD event and PCE variables.

	Hispanic/Latino Background				Genetic Ancestry		
	Hispanics Total	Mexican Hispanics	Caribbean Hispanics	Other Hispanics	>Median European Ancestry	>Median African Ancestry	>Median Amerindian Ancestry
Mean Age (SD), years	58 (9)	58 (9)	57 (9)	59 (8)	59 (9)	57 (8)	58 (8)
Female Gender, n ( %)	505 (52 %)	256 (9 %)	144 (51 %)	105 (59 %)	232 (50 %)	237 (51 %)	219 (53 %)
Mean Total cholesterol level (SD), mg/dL	197 (32)	200 (33)	194 (30)	196 (33)	198 (30)	196 (32)	198 (34)
Mean HDL-cholesterol level (SD), mg/dL	48 (13)	47 (13)	49 (13)	50 (94)	48 (13)	48 (13)	47 (12
Triglycerides, mg/dL	144 (69)	155 (70)	128 (63)	137 (71)	143 (69)	136 (67)	154 (68)
Blood Glucose (SD), mmol/L	91 (12)	91 (12)	91 (11)	90 (11)	91 (12)	91 (11)	91 (12)
Mean Systolic Blood Pressure (SD), mmHg	123 (21)	123 (21)	122 (19)	124 (11)	122 (20)	123 (20)	124 (22)
Mean Diastolic Blood Pressure (SD), mmHg	72 (10)	71 (11)	74 (9)	71 (10)	72 (10)	73 (10)	71 (10)
Antihypertensive Use Prevalence, n ( %)	222 (23 %)	93 (18 %)	88 (31 %)	41 (23 %)	118 (25 %)	128 (28 %)	74 (18 %)
Mean Body Mass Index (SD), kg/m^2^	29 (5)	30 (5)	29 (5)	28 (4)	29 (5)	29 (5)	30 (5)
Smoking Prevalence, n ( %)	148 (15 %)	67 (13 %)	59 (21 %)	22 (12 %)	78 (17 %)	82 (18 %)	53 (13 %)
No Health Insurance, n ( %)	200 (21 %)	128 (26 %)	16 (6 %)	58 (33 %)	73 (16 %)	75 (16 %)	124 (30 %)
Income <$25,000, n ( %)	414 (43 %)	218 (43 %)	109 (38 %)	87 (49 %)	182 (39 %)	197 (43 %)	193 (47 %)
Income >$100,000, n ( %)	24 (2 %)	14 (3 %)	5 (2 %)	5 (3 %)	15 (3 %)	8 (2 %)	9 (2 %)
Education beyond high school, n ( %)	367 (38 %)	180 (36 %)	116 (41 %)	71 (40 %)	185 (40 %)	170 (37 %)	133 (32 %)
Observed no. of ASCVD events*	54	33	13	8	32	20	26
Predicted no. of ASCVD events (NHW)⁎⁎	78	42	22	13	40	37	33
Predicted no. of ASCVD events (NHB)⁎⁎	81	43	24	15	41	39	34
Mean follow-up duration in years (min, max)	9.1 (0.3–10.1) years	9.1 (0.3–10.1) years	9.4 (0.7–10.1) years	9.0 (0.9–10.1) years	9.2 (0.3–10.1) years	9.2 (0.7–10.1) years	8.9 (0.6–10.1) years

⁎ Observed events from 10-year KM event probability times number of participants (rounded to nearest whole number).

⁎⁎ Predicted events from mean PCE score times number of participants (rounded to nearest whole number).

**Fig. 2:**
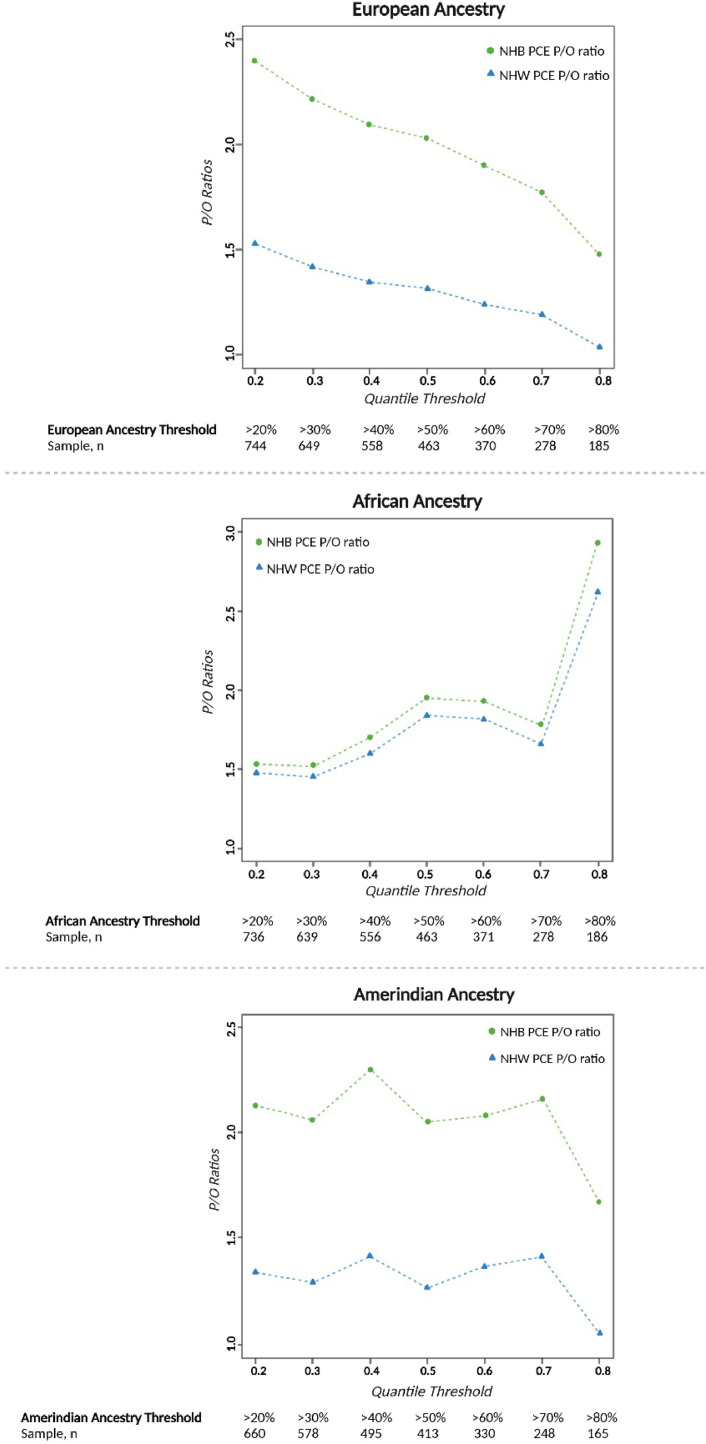
Predicted to Observed (P/O) ratios, using the Non-Hispanic Whites (NHW) and Non-Hispanic Blacks (NHB) Pooled Cohort Equation (PCE), among different quantile thresholds of African, Amerindian and European ancestry in MESA participants aged 45–75 years.

Overall, higher P/O ratios were observed among Hispanics/Latinos of greater than median African genetic ancestry compared to Hispanics/Latinos of greater than median European genetic ancestry and greater than median Amerindian genetic ancestry (Fig. 1B)**.** This overestimation was observed across all the three risk categories (low & borderline, intermediate and high-risk) and while using both the NHW and NHB PCE. Comparing the NHW PCE and the NHB PCE, we observed that the NHB PCE overestimated the P/O ratios for individuals with greater than median African, Amerindian, and European genetic ancestry within the low and borderline-risk group. Conversely, the NHW PCE overestimated P/O ratios for those with greater than median African, Amerindian, and European genetic ancestry in the intermediate-risk and high-risk categories, with the exception of individuals in the high-risk group with greater than median European ancestry (Fig. 1B)**.** Harrell’s C-statistic showed no statistically significant difference within groups (Table 2)**.** Both PCEs performed better (P/O ratios converging toward 1.0) in individuals with increasing percentages of European genetic Ancestry and performed worse (P/O ratios diverging from 1.0) with increasing percentages of African genetic Ancestry (Fig. 2)**.** Predictability was better at increasing thresholds of African and European genetic ancestry compared to Amerindian genetic ancestry using both NHW and NHB PCE (Fig. 2)**.**

**Table 2:** Risk Discrimination expressed as Harrell’s C-statistic (C-Stat) within the different Hispanic/Latino background and Genetic Ancestry groups.

Group	PCE	Harrel C-stat	p-value
Hispanic/Latino background group			
Mexican (MX)	NHW	0.737	0.838
	NHB	0.725	
Caribbean (CA)	NHW	0.684	0.107
	NHB	0.703	
Other Hispanic (O)	NHW	0.739	0.486
	NHB	0.740	
Genetic ancestry			
African Ancestry	NHW	0.719	0.099
	NHB	0.721	
European Ancestry	NHW	0.754	0.486
	NHB	0.755	
Amerindian Ancestry	NHW	0.718	0.636
	NHB	0.713	

NHW – Non-Hispanic White; NHB – Non-Hispanic Black; PCE – Pooled cohort equation.

The plot shows the P/O mean for those above each quantile threshold of given African, Amerindian and European genetic ancestry

## 4 Discussion

Our study included MESA Hispanics stratified by Hispanic/Latino background groups and by greater than median African, Amerindian, and European genetic ancestry. Among all MESA Hispanics, the PCE overestimated ASCVD events using NHB PCE (P/O ratio of 1.50) and NHW PCE (P/O ratio of 1.45). When disaggregating all MESA Hispanics into background groups (MX, CA, and O), we observed a lower overestimation among MX and a higher overestimation among CA and O compared to all MESA Hispanics.

CVD is the leading cause of mortality in the US, accounting for nearly a million deaths in 2020 [17]. Studies have shown the overall prevalence of ASCVD in the US to be 18.3 to 24 million among adults 21 years old and above [18,19], affecting about 1.5 % of Hispanic/Latino adults ≥ 20 years old [20]. This prevalence is expected to increase with the growing US population, particularly among Hispanics/Latinos [21]. Because the risk factors for ASCVD may not be readily apparent among seemingly healthy adults, accurate ASCVD risk prediction and primary prevention become extremely important, especially among populations underrepresented in research, such as Hispanics/Latinos.

Hispanics/Latinos are a highly admixed population, and studies have reported imprecise risk estimation using the PCE among Hispanic/Latino individuals [8,22,23]. Hispanics' heterogeneity, which has historically been overlooked, is important when estimating ASCVD risk because it allows us to observe how factors such as ethnic background and genetic admixture impact ASCVD risk estimation. It is therefore essential to account for the substantial heterogeneity in national origin and cultural background among Hispanics/Latinos, which spans over 20 countries [24]. These intersecting dimensions of identity influence health outcomes through distinct sociocultural, behavioral, environmental, and structural exposures [[25], [26], [27]]. For instance, despite a higher prevalence of cardiometabolic risk factors among Hispanic/Latino individuals compared to non-Hispanic Whites [5], some reports demonstrate lower all-cause and cardiovascular mortality in this population —a phenomenon commonly described as the "Hispanic paradox"[3]. These complex interactions between ancestry, culture, and social determinants of health underscore the need for disaggregated analyses and culturally informed risk prediction strategies, as the application of generalized models may inadequately capture within-group differences and potentiate misclassification.

Our results are similar to two prior studies that observed ASCVD risk overestimation among disaggregated Hispanic/Latino background groups [8,22]. However, neither of these studies examined ASCVD risk across strata defined by African, Amerindian, and European genetic ancestry quantiles. Our study findings showed higher P/O ratios among Hispanics/Latinos of greater than median or increasing proportions of African genetic ancestry and CA compared to Hispanics/Latinos of greater than median or increasing proportions of European genetic ancestry and MXs, respectively. Differences in the P/O ratios were observed when using both the NHB and NHW PCE. While the differences in baseline characteristics across Hispanic/Latino subgroups (like smoking and health insurance) may impact the PCE performance across Hispanic/Latino subgroups, there is a need to think through other factors beyond clinical and socio-demographic factors that may be driving these differences in a heterogeneous population such as Hispanics/Latinos.

After aggregating our Hispanic/Latino population into quantile thresholds of African, Amerindian, and European genetic ancestry, both PCEs performed better with increasing quantiles of European ancestry and performed worse with increasing quantiles of African ancestry. Predictability was better across thresholds of African and European genetic ancestry than Amerindian genetic ancestry using NHW and NHB PCE. Furthermore, we observed at the highest quantile of African genetic ancestry a sharp increase in overestimation for NHW and NHB PCEs. Using the NHW PCE, the P/O ratio markedly increased from 1.6, at the 60 % African ancestry quantile threshold, to 2.6 at the 70 % threshold. Similarly, using the NHB PCE, the P/O ratio increased from 1.75, at the 60 % African ancestry quantile threshold, to 2.9 at 70 % threshold. This finding suggests that among Hispanics/Latinos, genetic ancestry may play a role in ASCVD risk estimation; notably, greater than median African genetic ancestry among Hispanics/Latinos may predispose to overestimation of ASCVD risk compared to greater than median European or Amerindian ancestry when using both PCE's.

Comparing across ASCVD risk categories, the PCE performed increasingly worse in the higher ASCVD risk categories among CA but not for MX and O. Furthermore, the greatest overestimation was observed among CA and Hispanics/Latinos with greater than median African genetic ancestry who were within the clinically actionable ASCVD risk groups (intermediate and high risk). It can be suggested that a predictive equation may benefit public health by prioritizing sensitivity over specificity to reduce the likelihood of false negatives. However, caution is necessary to avoid exaggerated overestimation especially among Hispanics/Latinos by including this population in future risk prediction equations.

The American College of Cardiology (ACC) and American Heart Association (AHA) has recommended using the NHB PCE for 10-year ASCVD risk estimation among Hispanic/Latino individuals if African American ancestry is present [7]. Our analysis further explores the extent of ASCVD risk (over)estimation for Hispanics/Latinos with above-median genetic African ancestry. Using the NHB PCE, we observed even greater overestimation (P/O ratio of 1.95) than the NHW PCE (P/O ratio of 1.84) among MESA Hispanics with greater than median African ancestry.

Hispanics/Latinos are genetically diverse, encompassing a blend of European, African, and Amerindian ancestry. Consequently, Hispanics/Latinos may self-identify as one or multiple races, decline to self-report race, or report race as unknown despite an individual having predominant African, European, or Amerindian ancestry. Thus, self-reported race can be particularly challenging among Hispanics/Latinos and has mainly remained unreported or inaccurate in large epidemiologic cohorts. Self-reported race may not accurately reflect an individual's true genetic ancestry [28,29], particularly for Hispanics/Latinos. This is not surprising since race is a social construct, and the social context of race in Latin America is not an absolute equivalent to the binary concept of race in the US.

Moreover, genetic ancestry may provide a complementary framework than self-reported Hispanic background group. While self-identified background groups reflect shared cultural and environmental experiences, they are genetically diverse. Our prior work in MESA and Hispanic Community Health Study/Study of Latinos (HCHS/SOL) consistently shows that genetic ancestry patterns correlate with specific Hispanic background groups [6,30]. Further appreciation of genetic ancestry can potentially impact risk-prediction analyses, resulting in more robust risk-prediction model accuracy [30]. This notion deserves further study.

Our study included genetic ancestry analysis to quantify the degree of ASCVD (over)estimation in relation to ancestry. While clinicians may not be able to quantify the degree of genetic ancestry during a clinic visit, our findings based on risk calibration and discrimination can help to provide some insight into the nuances in ASCVD risk estimation among Hispanics/Latinos. Relying on clinical risk prediction models derived from populations that do not adequately represent the major demographic groups in the U.S. can lead to biased risk estimation and suboptimal care. Such models may fail to capture important variations in genetic background, environmental exposures, and sociocultural factors that influence disease risk. This lack of representativeness undermines the accuracy and clinical utility of cardiovascular risk assessments, particularly for historically underrepresented groups. Our study highlights the need for more equitable and representative approaches to cardiovascular prevention strategies and treatment approaches.

This study is one of the first studies to examine the performance of the PCE among disaggregated Hispanics/Latinos across African, Amerindian, and European genetic ancestry. Our study is not free of limitations. First, while study results may suggest that genetic ancestry, a biological variable, may have been used as a surrogate for self-reported race (a social construct), we do not know if genetic ancestry is a proxy for self-reported race in estimating ASCVD risk using the PCE among Hispanics/Latinos. Incorporating both genetic ancestry and culturally relevant factors in future prediction models may improve the precision and equity of cardiovascular risk assessment in Hispanics/Latinos, the largest ethnic minority population in the US, as broad ethnic labels often obscure meaningful genetic variation and overlook important subgroup-specific social determinants of health. Moreover, the intersection of genetic ancestry and ethnic background on ASCVD risk might be further impacted by other factors such as acculturation, social determinants of health (SDOH), and socioeconomic status. As such, individual and area-level SDOH risk factors may affect PCE calibration but not discrimination among Hispanics/Latinos when added to the PCE [31]. It is still unknown if the addition of social deprivation index [SDI), such as in the Predicting Risk of Cardiovascular Disease EVENTs (PREVENT) equation [32] and/or acculturation could improve the performance of the PCE among Hispanics/Latinos but that is the subject of ongoing research and beyond the scope of this study. We used the PCE since it is the recommended tool for ASCVD risk estimation prior to statin and blood pressure treatment, as per the recent guidelines [1]. Few events were recorded across each stratum of Hispanic/Latino background groups/genetic ancestry, and the majority (91 %) of Mexican Hispanics/Latinos were males which may decrease the power and generalizability of the study, respectively. Lastly, it is possible that MESA participants are healthier than the general population, and findings of overestimation of ASCVD risk using the PCE may potentially reflect healthy volunteer bias.

## 5 Conclusions

Both the NHW and NHB Pooled Cohort Equations overestimated the 10-year ASCVD risk among Hispanics/Latinos, as evidenced by predicted-to-observed ratios greater than 1.0. Disaggregating Hispanics/Latinos by background and genetic ancestry led to variability in PCE performance, with greater overestimation of ASCVD risk for those of Caribbean background and those with greater than median African genetic ancestry. Novel risk prediction models should consider stratifying Hispanics/Latinos by background groups/genetic ancestry in future predictive models.

## Sources of funding

This research was supported by contracts HHSN268201500003I, N01‐HC‐95159, N01‐HC‐95160, N01‐HC‐95161, N01‐HC‐95162, N01‐HC‐95163, N01‐HC‐95164, N01‐HC‐95165, N01‐HC‐95166, N01‐HC‐95167, N01‐HC‐95168, and N01‐HC‐95169 from the National Heart, Lung, and Blood Institute and by grants UL1‐TR‐000040 and UL1‐TR‐001079 from the National Center for Research Resources.

## Disclosures

None

Fig. 1A, Fig. 1B, Fig. 2, Table 1, Table 2

**Figure:**
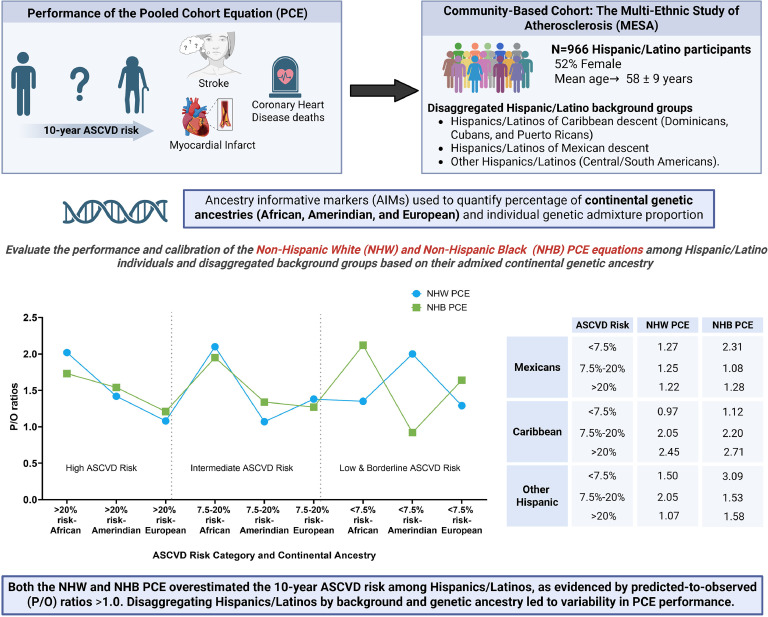
Central Illustration.

**Fig. 1A:**
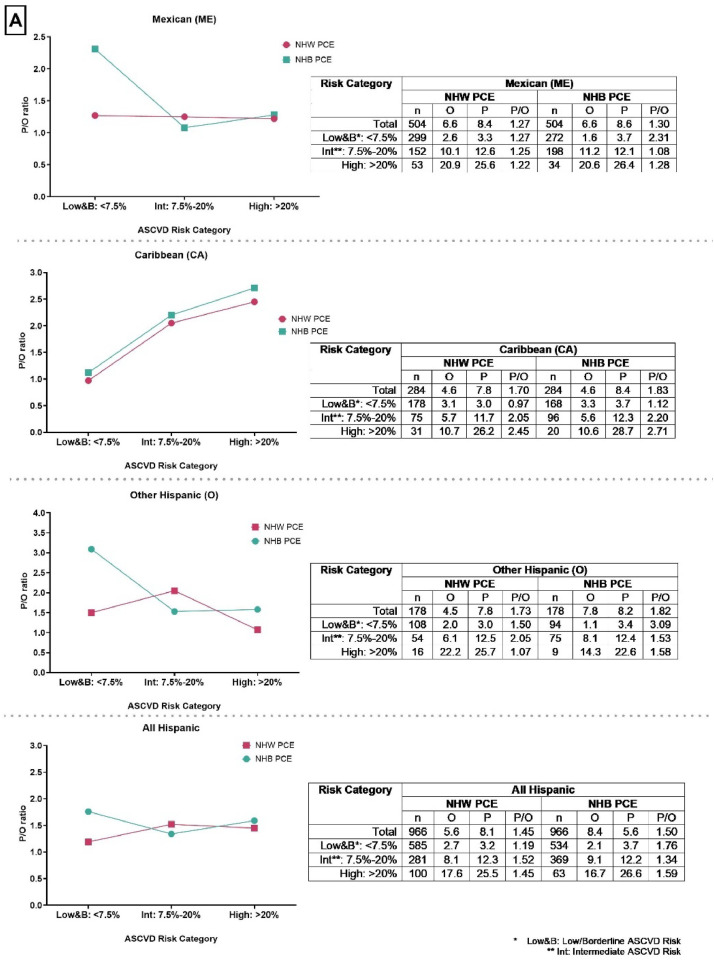
Pooled Cohort Equation (PCE) risk calibration expressed as predicted (P) to observed (O) ratios (P/O) across the different categories of ASCVD risk among MESA Hispanics/Latinos disaggregated by Ethnic Background.

**Fig. 1B:**
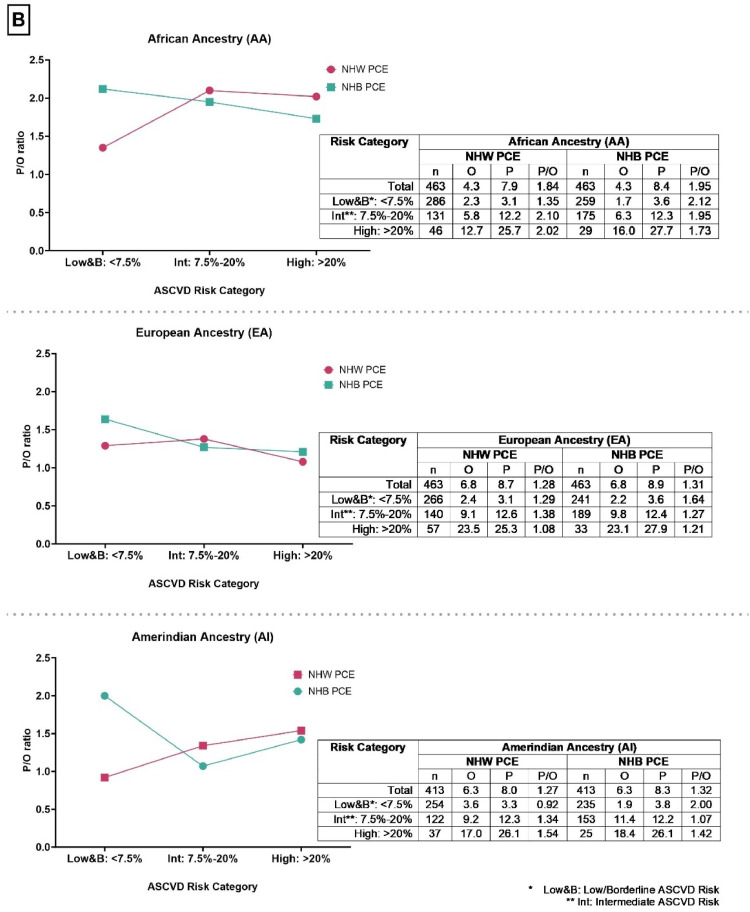
Pooled Cohort Equation (PCE) risk calibration expressed as predicted (P) to observed (O) ratios (P/O) across the different categories of ASCVD risk among MESA Hispanics/Latinos disaggregated by Genetic Ancestry.

## CRediT authorship contribution statement

**Bede N. Nriagu:** Writing – review & editing, Writing – original draft, Visualization, Methodology, Conceptualization. **Karen Flores Rosario:** Writing – review & editing, Conceptualization. **Spencer Hansen:** Writing – review & editing, Formal analysis. **Priscilla Duran Luciano:** Writing – review & editing, Visualization. **Parag Joshi:** Writing – review & editing. **Anurag Mehta:** Writing – review & editing. **Amit V. Khera:** Writing – review & editing. **Robert Kaplan:** Writing – review & editing. **Tamar Sofer:** Writing – review & editing. **Robyn L. McClelland:** Writing – review & editing. **Carlos J. Rodriguez:** Writing – review & editing, Supervision, Conceptualization.

## Declaration of competing interest

The authors declare that they have no known competing financial interests or personal relationships that could have appeared to influence the work reported in this paper.
